# LINC01133 as ceRNA inhibits gastric cancer progression by sponging miR-106a-3p to regulate APC expression and the Wnt/β-catenin pathway

**DOI:** 10.1186/s12943-018-0874-1

**Published:** 2018-08-22

**Authors:** Xian-Zi Yang, Tian-Tian Cheng, Qing-Jun He, Zi-Ying Lei, Jun Chi, Zhen Tang, Quan-Xing Liao, Hong Zhang, Li-Si Zeng, Shu-Zhong Cui

**Affiliations:** 1 Department of Medical Oncology, Affiliated Cancer Hospital & Institute of Guangzhou Medical University, Guangzhou, 510095 China; 2 Department of Abdominal Surgery, Affiliated Cancer Hospital & Institute of Guangzhou Medical University, Guangzhou, 510095 China; 3 Department of Endoscopy and Laser, Sun Yat-Sen University Cancer Center, Guangzhou, 510060 China; 4 State Key Laboratory of Oncology in South China, Collaborative Innovation Center for Cancer Medicine, Sun Yat-Sen University Cancer Center, Guangzhou, 510060 China

**Keywords:** Gastric cancer, LINC01133, ceRNA, miR-106a-3p, Progression, APC, Wnt/β-catenin pathway

## Abstract

**Background:**

Gastric cancer (GC) is a common malignancy and frequent cause of cancer-related death. Long non-coding RNAs (lncRNAs) have emerged as important regulators and tissue-specific biomarkers of multiple cancers, including GC. Recent evidence has indicated that the novel lncRNA LINC01133 plays an important role in cancer progression and metastasis. However, its function and molecular mechanism in GC remain largely unknown.

**Methods:**

LINC01133 expression was detected in 200 GC and matched non-cancerous tissues by quantitative reverse transcription PCR. Gain- and loss-of-function experiments were conducted to investigate the biological functions of LINC01133 both in vitro and in vivo. Insights into the underlying mechanisms of competitive endogenous RNAs (ceRNAs) were determined by bioinformatics analysis, dual-luciferase reporter assays, quantitative PCR arrays, TOPFlash/FOPFlash reporter assay, luciferase assay, and rescue experiments.

**Results:**

LINC01133 was downregulated in GC tissues and cell lines, and its low expression positively correlated with GC progression and metastasis. Functionally, LINC01133 depletion promoted cell proliferation, migration, and the epithelial–mesenchymal transition (EMT) in GC cells, whereas LINC01133 overexpression resulted in the opposite effects both in vitro and in vivo. Bioinformatics analysis and luciferase assays revealed that miR-106a-3p was a direct target of LINC01133, which functioned as a ceRNA in regulating GC metastasis. Mechanistic analysis demonstrated that miR-106a-3p specifically targeted the adenomatous polyposis coli (APC) gene, and LINC01133/miR-106a-3p suppressed the EMT and metastasis by inactivating the Wnt/β-catenin pathway in an APC-dependent manner.

**Conclusions:**

Our findings suggest that reduced expression of LINC01133 is associated with aggressive tumor phenotypes and poor patient outcomes in GC. LINC01133 inhibits GC progression and metastasis by acting as a ceRNA for miR-106a-3p to regulate APC expression and the Wnt/β-catenin pathway, suggesting that LINC01133 may serve as a potential prognostic biomarker and anti-metastatic therapeutic target for GC.

**Electronic supplementary material:**

The online version of this article (10.1186/s12943-018-0874-1) contains supplementary material, which is available to authorized users.

## Background

Gastric cancer (GC) is the fourth most common cancer and the third most frequent cause of cancer-related death worldwide, with the geographically highest incidence in East Asia (mainly China and Japan) [1]. Due to lack of symptoms in the early stages, most patients with GC are diagnosed with advanced disease or distant metastasis [2]. Despite important advances in diagnosis and therapeutic strategies in the past several decades, the prognosis for advanced-stage patients is still dismal [3, 4]. The complexity and multifactorial nature of the pathogenic and metastatic mechanism of GC are considered major obstacles in promoting the survival of patients with this disease [5]. Therefore, there is a pressing need to identify key molecular events underlying the progression and metastasis of GC in order to improve diagnosis and prognosis.

Long noncoding RNAs (lncRNAs) are a class of RNA polymerase II transcripts greater than 200 nucleotides in size [6]. In recent years, emerging evidence has implicated lncRNAs in multiple biological functions and they are thought to regulate multistep biological processes in various diseases, particularly cancer [7, 8]. Unlike the regulatory mechanisms of microRNAs (miRNAs), no common mechanism for the role of lncRNAs has been reported [9]. However, studies have shown that several lncRNAs fulfill their roles by “sponging” miRNAs and competitively inhibiting their biological functions [10, 11]. As a novel lncRNA, LINC01133 was first reported to be overexpressed in lung squamous cell cancer in 2015 [12]. A positive correlation was found between high LINC01133 expression in patients and poor prognosis, and LINC01133 knockdown led to inhibition of the proliferation and invasion of lung cancer and osteosarcoma cells [12–14]. Nevertheless, some studies have shown low LINC01133 expression in colorectal cancer (CRC), and LINC01133 overexpression was found to inhibit CRC cell metastasis by binding and blocking serine/arginine-rich splicing factor 6 function [9, 15]. The expression levels of LINC01133 vary across different types of cancer, suggesting that there is tissue-specific regulation of its expression. However, the expression and function of LINC01133 in GC remain unknown.

The epithelial-mesenchymal transition (EMT), a key step in cancer metastasis, is marked by the loss of epithelial characteristics and acquisition of mesenchymal characteristics [16, 17]. Emerging evidence has suggested that the Wnt/β-catenin pathway induces the EMT to maintain the integrity of epithelial cells as well as tight/adherent junctions [18]. Wnt ligands bind to Frizzled receptor complexes and activate Frizzled, which stabilizes cytoplasmic β-catenin protein by inhibiting the protein destruction complex (adenomatous polyposis coli [APC], axin, GSK3β, and casein kinase 1). The downregulation of APC contributes to nuclear accumulation of β-catenin, which subsequently interacts with the lymphoid enhancing factor/T cell factor (TCF) transcription factor to activate the transcription of target genes including c-Myc, cyclin D1, and matrix metalloproteinase 7 (MMP7) [19]. Indeed, overactivation of the canonical Wnt pathway was observed in 30–50% of GC tissues and cell lines [20, 21], and disruption of Wnt signaling prevented metastatic activity in GC cells [22]. However, it remains unclear whether and how lncRNAs are involved in the metastasis of GC by regulating the Wnt/β-catenin pathway. Therefore, in this study, we investigated the clinical significance of LINC01133 expression in GC, and obtained insights into the function and mechanisms underlying LINC01133 regulation of GC progression and metastasis.

## Methods

### GC patients and tissue specimens

A total of 200 GC tissues and pair-matched normal gastric epithelial tissues were obtained from GC patients who underwent radical surgery at Sun Yat-sen University Cancer Center (Guangzhou, China) from 2007 to 2011. No patient was given radiotherapy or chemotherapy before surgery. All cases were independently diagnosed histologically by two experienced pathologists and staged according to the TNM staging of the American Joint Committee on Cancer (AJCC 7th ed., 2010). All tissue samples were immediately frozen in liquid nitrogen after resection from GC patients and stored at − 80 °C for RNA extraction. This study, which used human cancer tissues, was approved by the Committee for Ethical Review of Research involving Human Subjects of Sun Yat-sen University, and informed consent was obtained from all patients.

### GC cell lines and culture conditions

Nine human GC cell lines (SUN-216, BGC-823, AGS, BGC-803, NUGC4, MKN74, MKN45, SGC-7901, and HGC-27) and a non-malignant gastric mucosal epithelial cell line GES-1 were purchased from the Cell Bank of the Chinese Academy of Sciences (Shanghai, China). Cells were cultured in RPMI-1640 medium supplemented with 10% fetal bovine serum (Hyclone, Logan, UT, USA). The human embryonic kidney (HEK) 293FT cell line was obtained from the American Type Culture Collection (Manassas VA, USA), and cultured and stored according to the provider’s instructions. All cell lines were maintained in a humidified cell incubator at 37 °C with an atmosphere of 5% CO_2_.

### RNA extraction and quantitative reverse transcription PCR (qRT-PCR)

Total RNA was isolated from patient tissues and cultured cells using TRIzol reagent (Invitrogen, Carlsbad, CA, USA) as per the manufacturer’s instructions. Total RNA (1 μg) was reverse transcribed to high-quality cDNA with the Moloney Leukemia Virus Reverse Transcriptase Kit (Promega, Madison, WI, USA). qRT-PCR was performed using the SYBR Green Mix (Promega) to detect expression of the long noncoding and coding genes; the results were normalized to GAPDH expression. The expression of the miRNAs in this study was measured using the All-in-One™ miRNA qRT-PCR Detection Kit (GeneCopoeia, Carlsbad, CA, USA), and qRT-PCR was performed on the ABI 7500 Fast Real-Time PCR System (Applied Biosystems, Waltham, MA, UK). Small RNA RNU6B (U48) was used as the endogenous control to normalize miRNA expression. Each sample was run in triplicate, and fold changes were calculated using the relative quantification 2^-△△CT^ method. The primer sequences used in this study are shown in Additional file 1: Table S1.

### Lentivirus production and cell transfection

The lentivirus-containing short hairpin RNA (shRNA) targeting LINC01133 was purchased from GenePharma (Shanghai, China), and the pLenti-GIII-CMV vector for LINC01133 overexpression was purchased from Applied Biological Materials (Richmond, Canada); both shRNAs were transfected into the GC cell lines. At 48 h post-transfection, the cells were selected with puromycin (2 μg/mL) for 2 weeks to construct cell lines with stable LINC01133 knockdown or overexpression. The transfection efficiency was verified by qRT-PCR. The has-miR-106a-3p mimic, hsa-miR-106a-3p inhibitor, and negative control (NC) oligonucleotides were obtained from Ribobio (Guangzhou, China). The pCMV-Neo-Bam-APC vector for upregulation of the APC gene was obtained from Addgene (Cambridge, MA, USA). GC cells were transfected with the abovementioned oligonucleotides and plasmids using Lipofectamine 3000 (Invitrogen) according to the manufacturer’s instructions.

### Cell proliferation and colony formation assays

For the cell proliferation assay, 24 h after transfection, tumor cells were seeded into 96-well plates and successively cultured for 7 days. Each well was added with 10 μL Cell Counting Kit-8 (CCK-8) solution (Dojindo, Tokyo, Japan) daily. Cell viability was evaluated by measuring the absorbance values at a wavelength of 450 nm. For the colony formation assay, tumor cells (500 cells per well) were seeded into 6-well plates 24 h after transfection. After 2 weeks of incubation, the cells were fixed in methanol and stained with 0.1% crystal violet. The colonies were counted using Quantity One software (Bio-Rad, Hercules, CA, USA). All experiments were repeated three times and the mean was calculated.

### Cell migration and wound healing assays

Cell migration ability was measured using transwell chambers (8-μm pore size; Corning Costar, Cambridge, MA, USA). For the transwell assay, cells suspended in serum-free RPMI-1640 medium were seeded into the upper chamber. The lower chamber contained RPMI-1640 medium supplemented with 20% serum, which served as a chemoattractant. After 24 or 48 h incubation, the filters were fixed in methanol and stained with 0.1% crystal violet. The upper faces of the filters were gently abraded, and the lower faces with cells migrated across the filters were imaged and counted under the microscope. For wound healing assays, cells were placed into 6-well plates and cultured until 100% confluence. An artificial scratch was created using a 200-μL pipette tip. At 0 and 24 h after culturing in serum-free medium, wound closure images were captured in the same field under magnification. Cell healing rates were calculated by the fraction of cell coverage across the line. These experiments were performed in triplicate and repeated three times.

### Immunofluorescence (IF) assay

Cells were seeded on glass coverslips in 24-well plates and grew overnight. Then cells were fixed in 4% paraformaldehyde (PFA) for 20 min and permeabilized with 0.25% Triton X-100 for 10 min. After blocked with 4% bovine serum albumin, the coverslips were incubated with primary antibody (4 °C, overnight) against E-cadherin or vimentin (Cell Signaling Technology, Danvers, MA, USA) followed by incubation with the fluorescent secondary antibody. Cellular nuclei were stained using 4′,6-diamidino-2-phenylindole dihydrochloride (DAPI; Life Technologies, Carlsbad, CA, USA). Images of the coverslips were observed under a confocal laser scanning microscope (FV1000; Olympus, Tokyo, Japan).

### Fluorescence in situ hybridization (FISH)

AGScells were fixed in 4% PFA for 15 min at room temperature and then permeabilized with 0.5% Triton X-100 for 15 min at 4 °C. Cells were incubated with digoxigenin (DIG)-labeled LINC01133 probe or Control-FISH probe mix for 4 h at 55 °C and briefly washed with 2 × saline-sodium citrate six times for 5 min. Horseradish peroxidase (HRP)-conjugated anti-DIG secondary antibodies (Jackson, West Grove, PA, USA) were used for detecting the signal, and nuclei were counterstained with DAPI. Images were obtained using an Olympus confocal laser scanning microscope.

### Luciferase reporter and TOPFlash/FOPFlash reporter assays

LINC01133 cDNA containing the predictive binding site of miR-106a-3p was cloned into the pmirGLO Dual-Luciferase miRNA Target Expression Vector (Promega) to form the reporter vector named pmirGLO-LINC01133-WT. The mutant LINC01133 containing point mutations of the miR-106a-3p seed region binding site, was specifically synthesized and inserted into the abovementioned vector, which was named pmirGLO-LINC01133-Mut. HEK-293FT cells were cultured into 96-well plates and co-transfected with pmirGLO-LINC01133–3’-UTR vectors including wild-type or mutant fragments and miR-106a-3p mimic, and the pmirGLO vector was used as the NC. To confirm the direct interaction of miR-106a-3p and APC, wild-type and mutant APC 3’-UTR fragments were amplified by qRT-PCR and cloned into the pmirGLO vector (Promega) using the one-step directed cloning kit (Novoprotein, Shanghai, China); the resultant vectors were termed APC-WT and APC-Mut, respectively. The miR-106a-3p mimic or inhibitor was co-transfected with APC-WT or APC-Mut vector into HEK-293FT cells using Lipofectamine 3000 (Invitrogen). At 48 h post-transfection, the luciferase assay was performed using the Dual Luciferase Reporter Assay System (Promega) according to the manufacturer’s instructions. Relative firefly luciferase activity was normalized to Renilla luciferase activity as a control for transfection efficiency. For the TOPFlash/FOPFlash reporter assay, cells were co-transfected with the Wnt/β-catenin signaling reporter TOPFlash/FOPFlash (Addgene, Cambridge, MA, USA), along with LINC01133 knockdown or overexpression vector, and/or miR-106a-3p inhibitor or the miRNA control. At 48 h post-transfection, GC cells were lysed, whereas HEK293FT cells were treated with 20 mM LiCl-conditioned medium for 6 h following cell lysis. The results are represented as normalized TOPFlash/FOPFlash values as previously described [23]. The primers used for vector construction are provided in Additional file 1: Table S1. These results are presented as the mean ± standard deviation (SD) of three replicates.

### miRNA- and metastasis-related gene validation by the quantitative PCR (qPCR) array

To validate the miRNAs, we first predicted all potential targeted miRNAs of LINC01133 using publicly available bioinformatic databases. According to these potential targeted miRNAs, the miProfile Customized miRNA qPCR Array (GeneCopoeia, Rockville, MD, USA) 384-well qPCR plate was used for miRNA validation. The miRNA primers were also provided by GeneCopoeia. Reverse transcription product (1 μL) was added to the 10 μL qPCR array reaction volumes according to the manufacturer’s recommendation. For validation of the metastasis-related genes, the ExProfile™ Human Metastasis Related Gene qPCR Array (GeneCopoeia) was also performed. Each 96-well PCR array contained 84 metastasis-related genes. The data were normalized to U48 or GAPDH by the 2^-△△CT^ method. *P* <  0.05 with a fold change greater than 2.0 was considered significant dysregulation. The primer sequences used in this study are shown in Additional file 1: Table S1.

### In vivo tumor formation and metastasis assays

Four-week-old immunodeficient BABL/c female nude mice were purchased and maintained under specific pathogen-free conditions. For the in vivo tumor formation assay, SGC-7901 cells transfected with lentivirus containing LINC01133 sequence or empty vector were separately injected into the right dorsal flank of BABL/c nude mice. The tumor volume was measured every 4 days. Four weeks after injection, the animals were sacrificed and the xenograft tumors were dissected and weighed for qRT-PCR and immunohistochemistry assays. For the tumor metastasis assay, the abovementioned cells were injected into nude mice via the tail vein. After 50 days, the mice were euthanized and the lungs were removed for immunohistochemistry and qRT-PCR assays. The images and number of metastatic nodules in the lungs were captured and counted under a dissecting microscope. All animal studies were conducted with the approval of the Institutional Animal Care and Use Committee of Sun Yat-sen University.

### Immunoblot analysis

Total proteins from cells were extracted using RIPA buffer (Cell Signaling Technology) supplemented with protease inhibitors and phosphatase inhibitors. Proteins of equal amounts (30 μg) were separated by sodium dodecyl sulfate polyacrylamide gel electrophoresis and transferred to polyvinylidene difluoride membranes. After blocking antigen, the membranes were incubated with primary antibodies and the corresponding secondary antibodies. Proteins were visualized with Western Lightning Chemiluminescence Reagent Plus (PerkinElmer, Waltham, MA, USA). The primary antibodies used in this assay included E-cadherin, vimentin, N-cadherin, β-catenin, Lamin B1, GAPDH (Cell Signaling Technology), and APC (Abcam, Cambridge, UK). HRP-conjugated goat anti-rabbit or anti-mouse IgG antibody (Abcam) was used as the secondary antibody. For extraction of cytoplasmic and nuclear proteins, BeyoECL Plus Nuclear and Cytoplasmic Protein Extraction Kits (Beyotime Institute of Biotechnology, Jiangsu, China) were utilized according to the manufacturer’s instructions.

### Immunohistochemical analysis

Immunohistochemical (IHC) staining was performed as previously described [24]. Briefly, the dissected tumors from the mouse model experiment were paraffin-embedded and cut into 4 μm thick sections. These sections were deparaffinized and rehydrated. After antigen retrieval, the sections were incubated with anti-Ki67 and anti-MMP-9 antibodies (Abcam) at 4 °C overnight. After incubation with HRP-conjugated secondary antibody (Dako, Glostrup, Denmark), the sections were counterstained with Mayer’s hematoxylin. The immunostaining score was independently evaluated by two pathologists.

### Statistical analysis

All statistical analyses were conducted using SPSS 23.0 (SPSS, Chicago, IL, USA) or GraphPad PrismV7 (GraphPad Prism, Inc., La Jolla, CA, USA). Each experiment was performed at least in triplicate, and data are presented as the mean ± SD of three independent experiments. Student’s *t*-test or one-way ANOVA was used to compare the means of two or three groups. The correlation between LINC01133 expression and clinicopathological variables was calculated by the chi-square test or Fisher’s exact test. The Kaplan-Meier method and log-rank test were employed to generate the survival curve and compare differences between survival curves, respectively. *P* values less than 0.05 were considered statistically significant.

## Results

### LINC01133 expression decreases in GC tissue and cell lines

**Fig. 1:**
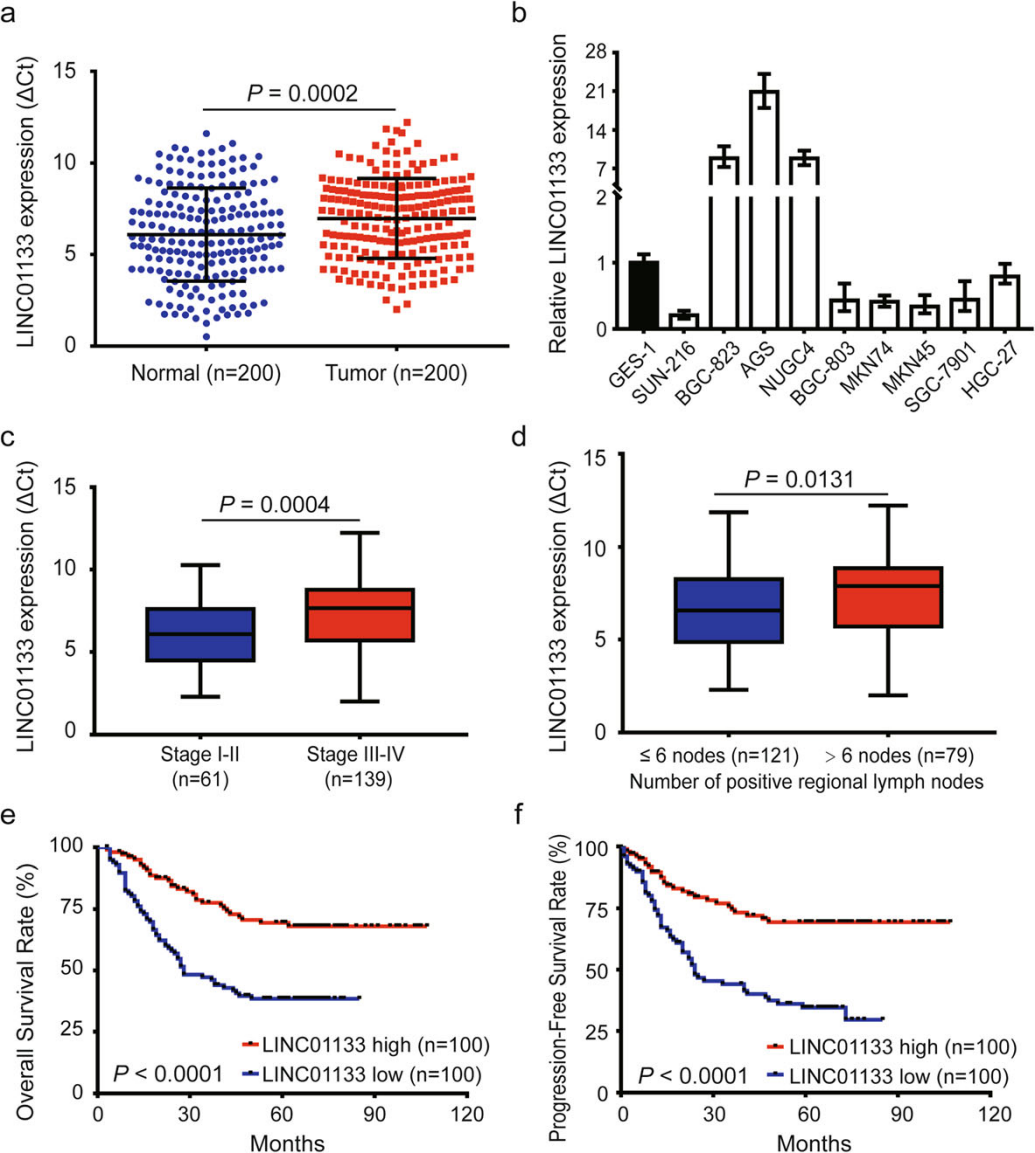
LINC01133 is downregulated in GC and negatively correlates with poor prognosis in GC patients. **a**. Relative expression of LINC01133 detected by qRT-PCR in 200 paired GC cancer tissues and matched normal tissues. Results are presented as Δ cycle threshold (ΔCt) in tumor tissues relative to normal tissues. **b** qRT-PCR analysis of the relative expression of LINC01133 in nine GC cell lines and the immortalized gastric normal mucosal cell line GES-1. **c**, **d** Relative expression of LINC01133 in GC with different TNM stage and different numbers of positive regional lymph nodes. **e**, **f** Kaplan-Meier plots of the OS and PFS of GC patients with high (*n* = 100) and low (*n* = 100) levels of LINC01133. Data are presented as the mean ± SD

We used qRT-PCR to determine the expression of LINC01133 in 200 paired GC tissues and matched normal tissues. As shown in Fig. 1a, the expression of LINC01133 in GC tissues was markedly decreased compared with normal tissues (*P* = 0.0002). Meanwhile, LINC01133 expression was significantly lower in six of the nine GC cell lines than in the GES-1 normal gastric epithelia cell line (Fig. 1b). In different clinical subgroups, LINC01133 expression was clearly reduced in patients with advanced TNM stage (stage III–IV) and lymphatic metastasis (number of positive regional lymph node > six nodes) (Fig. 1c, d). Although LINC01133 expression was negatively correlated with larger tumor, infiltration of peritumoral tissues, distant metastasis, and peritoneum dissemination, there were no statistically significant differences among these subgroups (Additional file 2: Figure S1a–d). Collectively, these clinical data suggest that the reduced expression of LINC01133 is frequently observed in GC and may be involved in GC progression and metastasis.

### Low expression of LINC01133 is positively associated with tumor progression and poor prognosis in GC patients

To reveal the clinical relevance of LINC01133 expression in GC patients, the median score (0.50) of relative LINC01133 expression was defined as the cutoff value for dividing all GC patients into high- and low-expression groups (Additional file 2: Figure S1e). Further analysis showed that low LINC01133 expression was significantly positively associated with larger tumor, advanced T stage, lymphatic invasion, advanced TNM stage, and infiltration of peritumoral tissues (Additional file 3: Table S2). To assess the prognostic value of LINC01133 expression, we analyzed the effects of LINC01133 status on overall survival (OS) and progression-free survival (PFS) in patients with this malignancy. Kaplan–Meier analysis demonstrated that patients with low LINC01133 expression had a much shorter 5-year OS (38.4% vs. 69.4%) and 5-year PFS (34.5% vs. 69.3%) than those with high LINC01133 expression (all *P* <  0.0001; Fig. 1e, f). Moreover, univariate analysis indicated that T stage, N stage, TNM stage, distant metastasis, infiltration of peritumoral tissues, peritoneum dissemination, and LINC01133 expression were noteworthy predictors of OS and PFS in GC patients (all *P* <  0.01, Table 1). Multivariate analysis showed that LINC01133 was an independent protective predictor of OS and PFS (Table 1). In addition, TNM stage, distant metastasis, and infiltration of the peritumoral tissues also were independent risk predictors of OS and PFS in GC patients (Table 1). Taken together, these data suggest that LINC01133 may serve as a promising biomarker for predicting outcome in GC patients.

**Table 1:** Univariate and multivariate Cox regression analysis of LINC01133 and survival in patients with gastric cancer

Clinical variables	Univariate analysis		*P* value	Multivariate analysis		*P* value
	HR	95% CI		HR	95% CI	
Overall Survival						
Gender (Male vs. Female)	0.776	0.500–1.203	0.257			
Age (≥60y vs. <60y)	0.978	0.639–1.496	0.918			
Tumor location (Down vs. Upper/Middle)	0.784	0.512–1.199	0.261			
Tumor size (≥5 cm vs. < 5 cm)	1.413	0.913–2.185	0.121			
Differentiation (Poor vs. Moderate/Well)	1.855	0.960–3.587	0.066			
T stage (T3-T4 vs. T1-T2)	4.126	1.512–11.259	**0.006**			
N stage (N2-N3 vs. N0-N1)	2.117	1.304–3.436	**0.002**			
TNM stage (III-IV vs. II-I)	3.756	2.040–6.918	**< 0.001**	2.637	1.405–4.951	**0.003**
Distant metastasis (Yes vs. No)	3.533	2.061–6.059	**< 0.001**	2.133	1.227–3.706	**0.007**
Infiltration of peritumoral tissues (Yes vs. No)	2.521	1.646–3.862	**< 0.001**	1.956	1.269–3.014	**0.002**
Peritoneum dissemination (Yes vs. No)	3.725	1.958–7.084	**< 0.001**			
LINC01133 expression (high vs. low)	0.381	0.244–0.597	**< 0.001**	0.517	0.328–0.815	**0.005**
Progression-Free Survival						
Gender (Male vs. Female)	0.812	0.518–1.273	0.364			
Age (≥60y vs. <60y)	0.805	0.518–1.250	0.333			
Tumor location (Down vs. Upper/Middle)	0.722	0.468–1.114	0.141			
Tumor size (≥5 cm vs. < 5 cm)	1.210	0.782–1.872	0.391			
Differentiation (Poor vs. Moderate/Well)	1.127	0.635–2.001	0.682			
T stage (T3-T4 vs. T1-T2)	5.979	1.887–18.945	**0.002**			
N stage (N2-N3 vs. N0-N1)	2.419	1.463–4.000	**0.001**			
TNM stage (III-IV vs. II-I)	3.511	1.941–6.351	**< 0.001**	2.493	1.352–4.595	**0.003**
Distant metastasis (Yes vs. No)	3.052	1.706–5.459	**< 0.001**	1.855	1.024–3.360	**0.042**
Infiltration of peritumoral tissues (Yes vs. No)	2.381	1.532–3.699	**< 0.001**	1.792	1.146–2.802	**0.011**
Peritoneum dissemination (Yes vs. No)	2.210	1.010–4.835	**0.047**			
LINC01133 expression (high vs. low)	0.345	0.217–0.547	**< 0.001**	0.460	0.287–0.736	**0.001**

NOTE: *TNM* tumour node metastasis. *HR* hazard ratio, *CI* confidence interval. The bold type represents *P* values smaller than 0.05

### Ablation of LINC01133 elicits the proliferation and migration of GC cells

**Fig. 2:**
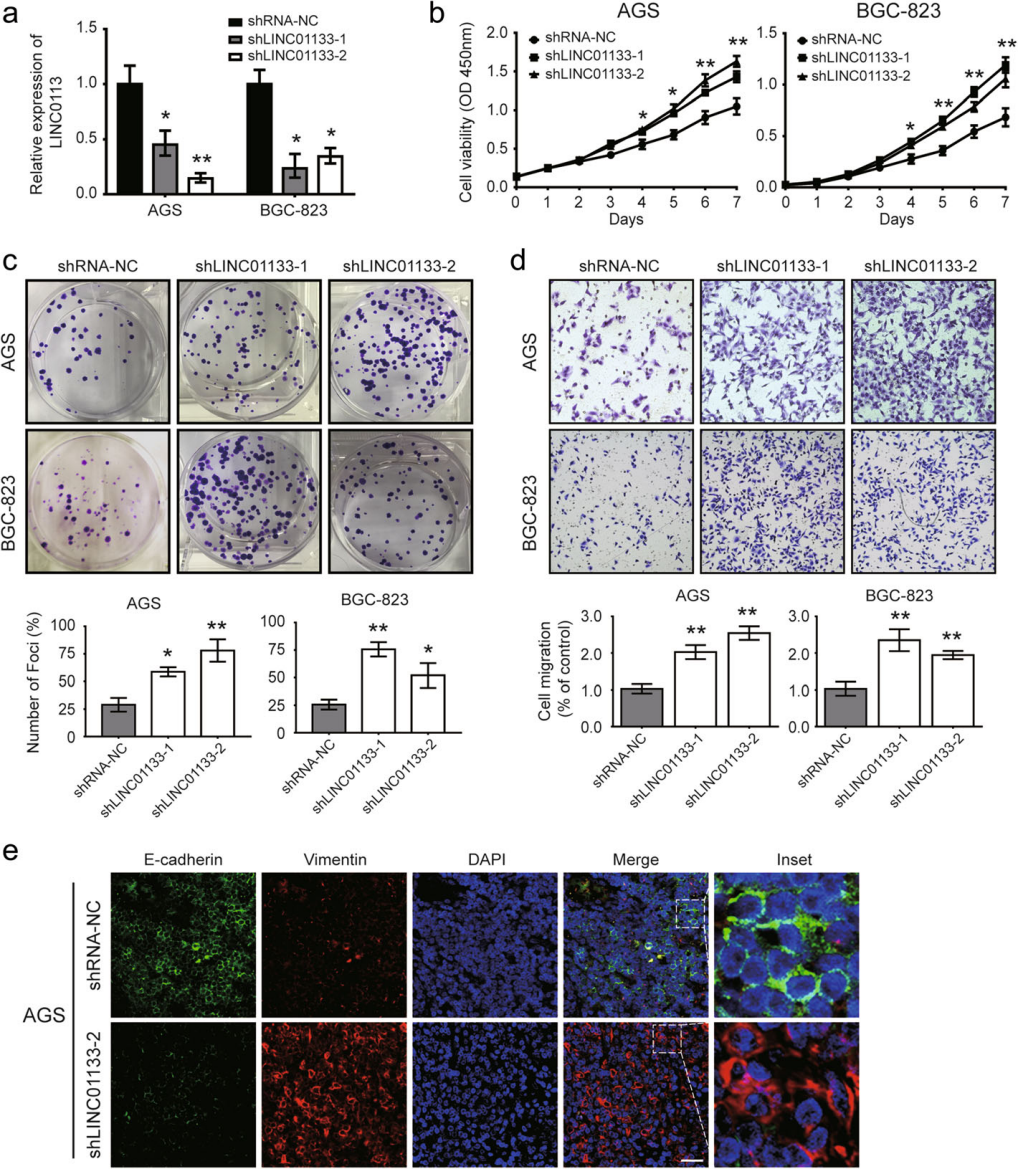
Reduced expression of LINC01133 promotes proliferation and migration and induces the EMT in GC cells. **a** qRT-PCR was conducted to verify the relative expression of LINC01133 in AGS and BGC-823 cells transfected with two independent shRNAs targeting LINC01133. **b** CCK-8 assay of AGS and BGC-823 cells after knockdown of LINC01133. **c**, **d** Representative results of the colony formation and transwell assays of AGS and BGC-823 cells after shLINC01133–1 or shLINC01133–2 transfection. **e** Representative images of IF micrographs of the subcellular localization and expression of E-cadherin (green) and vimentin (red). Nuclei were counterstained with DAPI (blue). Scale bars represent 50 μm. For all quantitative results, the data are presented as the mean ± SD from three independent experiments. ^*^P <  0.05 and ^**^P <  0.01

Since LINC01133 expression is downregulated and negatively correlates with GC progression and metastasis, we used loss-of-function experiments to determine whether it influences GC cell proliferation and migration. AGS and BGC-823 cell lines with the highest level of LINC01133 were selected for in vitro experiments (Fig. 1b). We used shRNA to silence LINC01133 expression, and effective knockdown of LINC01133 in both cell lines were verified by qRT-PCR (Fig. 2a). CCK-8 and colony formation assays were subsequently performed, and the results indicated that ablation of LINC01133 promoted cell growth and colony formation (Fig. 2b, c). On the other hand, LINC01133 knockdown significantly enhanced the cell migratory capacity and wound healing (Fig. 2d and Additional file 4: Figure S2a). Interestingly, the IF assay showed that loss of LINC01133 changed the morphology of the AGS cells from the condensed type into the dispersed type, which was accompanied by the increased expression of the mesenchymal marker vimentin and decreased expression of the epithelial marker E-cadherin (Fig. 2e). These data confirm that the reduced expression of LINC01133 promoted GC growth and metastasis in vitro.

### LINC01133 inhibits GC metastasis and EMT in vitro and in vivo

**Fig. 3:**
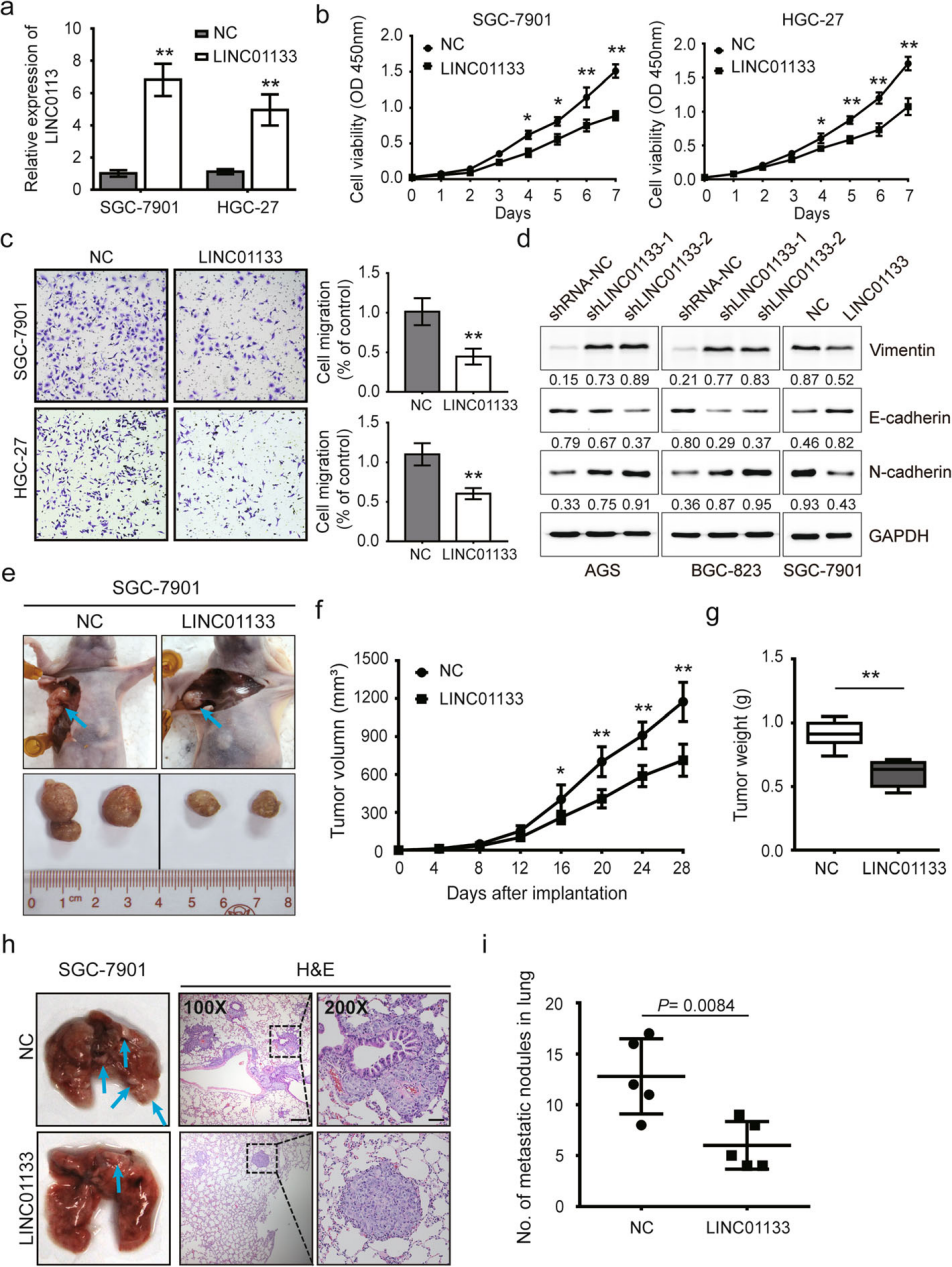
LINC01133 inhibits proliferation, migration and EMT in vitro and in vivo. **a** Relative expression of LINC01133 confirmed by qRT-PCR in SGC-7901 and HGC-27 cells with LINC01133 overexpression. **b**, **c** Proliferation and migration assays of SGC-7901 and HGC-27 cells with LINC01133 overexpression by the CCK-8 assay (**b)**, and transwell assay (**c)**. **d** Immunoblot assay to evaluate the expression of vimentin, E-cadherin, and N-cadherin proteins in AGS and BGC-823 cells after LINC01133 knockdown or in SGC-7901 cells after LINC01133 overexpression. The reference protein GAPDH was used as an internal control. Numbers shows the ratio of target protein/GAPDH (arbitrary unit). **e** The right armpit was injected with SGC-7901 cells transfected with LINC01133 expression vector or empty vector in upper panel. Representative images of xenograft tumors are indicated in the bottom panel. **f**, **g** Tumor volume and weight of the xenograft in LINC01133 overexpression groups and control group. **h** Left panel: representative images of lung metastatic nodules of LINC01133 overexpression groups and control group are indicated by blue arrows. Representative hematoxylin and eosin (H&E) staining results of corresponding lung metastatic nodules are shown in right panel. **i** Statistical analysis of numbers of metastatic nodules in the lung. Error bars: mean ± SD from three independent experiments. ^*^P <  0.05 and ^**^P <  0.01

We also constructed the pLenti-GIII-CMV-2A-puro expression vector to overexpress LINC01133 in SGC-7901 cells and HGC-27. LINC01133 expression was significantly upregulated in pre-LINC01133-transfected cells (Fig. 3a). Results from the CCK-8, colony formation, and transwell assays demonstrated that cell proliferation, colony formation, and migration were significantly repressed after LINC01133 overexpression (Fig. 3b, c and Additional file 4: Figure S2b). We performed western blotting to determine whether LINC01133 affected the EMT process of GC cells. The results showed that knockdown of LINC01133 elevated the expression of vimentin and N-cadherin and decreased E-cadherin expression. Conversely, overexpression of LINC01133 had the opposite effects on the EMT (Fig. 3d). To evaluate the proliferative and metastatic potential of LINC01133 in vivo, SGC7901 cells with stable LINC01133 expression were subcutaneously injected into female nude mice (Fig. 3e). As expected, smaller volume and lighter weight of xenograft tumors were observed in SGC-7901-LINC01133 group compared with NC group (Fig. 3f, g). Meanwhile, we intravenously injected the abovementioned cells into nude mice to construct a lung metastasis model. Consistent with the in vitro data, the numbers of metastatic nodules in the lung were fewer in SGC-7901-LINC01133 group than in NC group (Fig. 3h, i). The IHC assay showed that in the dissected tumors, LINC01133 overexpression inhibited the immunostaining of Ki-67 and MMP-9 (Additional file 4: Figure S2c). In lung metastases, the mRNA levels of Ki-67 and MMP-9 were decreased in SGC-7901-LINC01133 group compared with NC group (Additional file 4: Figure S2d). Therefore, The results of the in vitro and in vivo experiments strongly suggest that LINC01133 is a novel tumor suppressor in GC progression and metastasis.

### LINC01133 acts as a competitive endogenous RNA for miR-106a-3p in GC

**Fig. 4:**
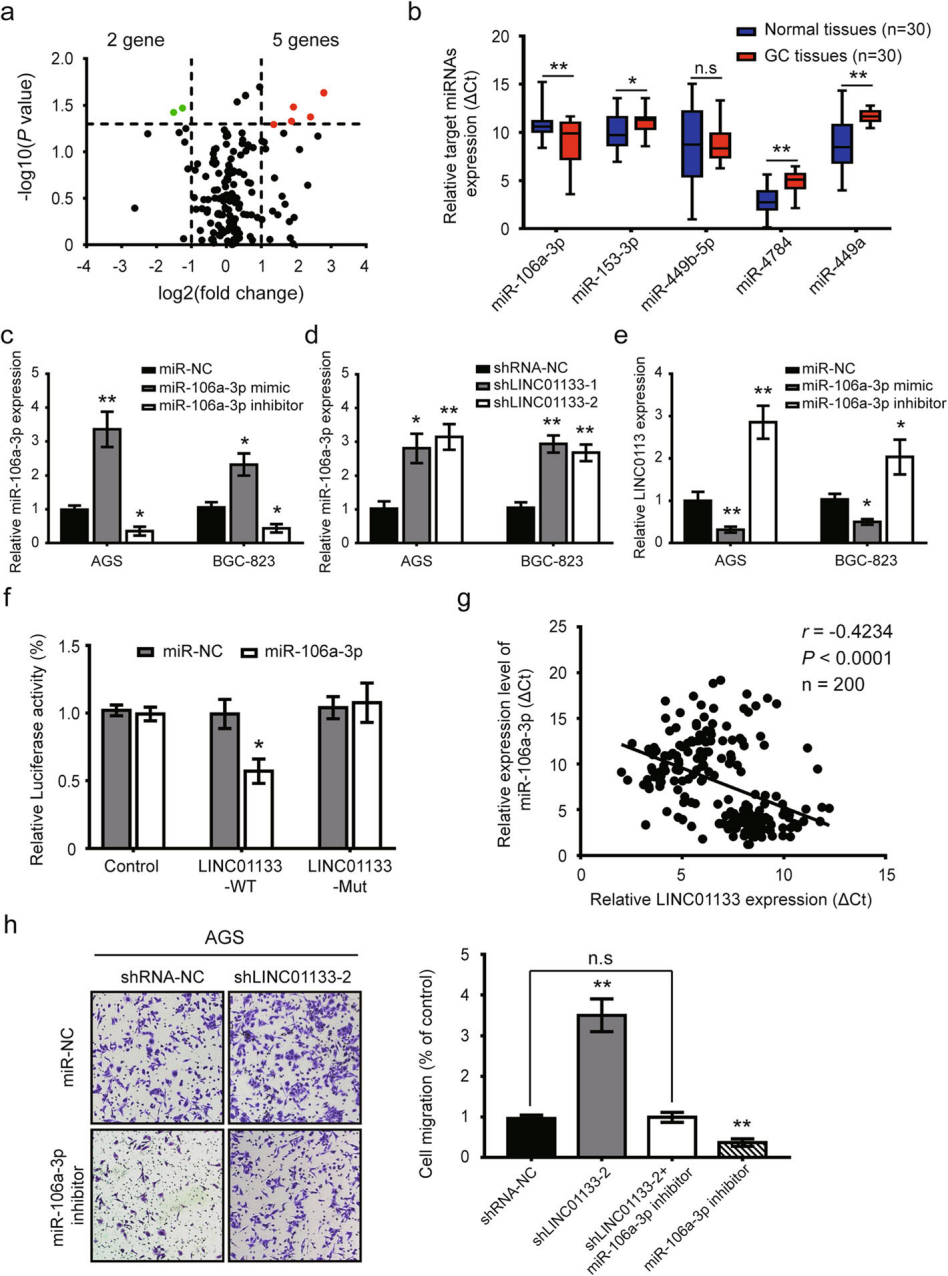
LINC01133 inhibited GC metastasis by directly targeting miR-106a-3p. **a** Volcano plot of different targeted miRNAs of LINC01133 on AGS cells transfected with shLINC01133–2 compared with cells transfected with scramble sequence (NC). Red marks, > 2-fold change (log2) and *P* <  0.05 (−log10); Green marks, < − 2-fold change (log2) and *P* <  0.05 (−log10). **b** qRT-PCR assay further confirmed the expression levels of five upregulated candidate miRNAs in 30 paired GC cancer tissues and matched normal tissues. **c**, **d** Relative expression of miR-106a-3p in AGS and BGC-823 cells transfected with miR-106a-3p mimic or inhibitor **c** or cells after transfection with shLINC01133 or scramble sequence (**d)**. **e** Relative expression of LINC01133 in AGS and BGC-823 cells transfected with miR-106a-3p mimic or inhibitor. **f** Relative luciferase activities of wild type (WT) and mutated (Mut) LINC01133 reporter plasmid in human embryonic kidney (HEK) 293FT cells co-transfected with miR-106a-3p mimic. **g** Correlation analysis between LINC01133 and miR-106a-3p expression in 200 GC tumor tissues. **h** Transwell assay following low expression of LINC01133 and/or inhibition of miR-106a-3p. Representative images (left panel) and quantifications (right panel) were shown. Error bars: mean ± SD. n.s, not significant, ^*^P <  0.05 and ^**^P <  0.01

To further explore the molecular mechanism of LINC01133 regulation in GC, we performed FISH assay to determine the subcellular localization of LINC01133. As shown in Additional file 5: Figure S3a, LINC01133 transcript was predominately localized in the cytoplasm, which was consistent with previous findings in CRC [9]. The most well-known mechanism of cytoplasmic lncRNAs is competitive endogenous RNAs (ceRNAs), which sponge various miRNAs to dampen their regulatory effects on target mRNAs [25]. Next, we collectively predicted 162 potential conjugated miRNAs using publicly available algorithms (Additional file 5: Figure S3b). Using a human custom miRNA qPCR array, we identified five upregulated miRNAs and two downregulated miRNAs in AGS cells with LINC01133 knockdown (Fig. 4a). Among the five upregulated miRNAs, we further verified that miR-106a-3p expression was significantly increased in 30 GC tissues compared with matched normal tissues (Fig. 4b).

To determine whether LINC01133 could function as a ceRNA for miR-106a-3p, we used qRT-PCR assay to evaluate miR-106a-3p expression in GC. The results showed that miR-106a-3p expression was significantly overexpressed in 200 paired GC samples (Additional file 5: Figure S3c) and miR-106a-3p expression was increased or decreased after AGS and BGC-823 cells were transfected with miR-106a-3p mimic or inhibitor, respectively (Fig. 4c). LINC01133 knockdown led to the significant upregulation of miR-106a-3p expression, whereas its overexpression resulted in the downregulation of miR-106a-3p (Fig. 4d, e). Bioinformatics tools further predicted two potential binding sites for miR-106a-3p in LINC01133 (Additional file 5: Figure S3d). To obtain direct evidence for the interaction between LINC01133 and miR-106a-3p, we subcloned wild-type (LINC01133-WT) and mutated (LINC01133-Mut) miR-106a-3p binding site into dual-luciferase reporters. Fig. 4f shows that the relative luciferase activity of LINC01133-WT in HEK293FT cells was obviously reduced after co-transfection of miR-106a-3p mimic, but did not change the activity of mutant vector, which suggest that miR-106a-3p is a direct target of LINC01133. Consistently, there was a negative correlation between LINC01133 and miR-106a-3p in GC tissues (Fig. 4g). As expected, the pro-metastatic activity of LINC01133 knockdown was reversed by co-transfection with miR-106a-3p inhibitor (Fig. 4h). Taken together, these data show that LINC01133 functions as a ceRNA for miR-106a-3p in the regulation of GC metastasis.

### LINC01133 inhibits GC migration by targeting miR-106a-3p/APC

**Fig. 5:**
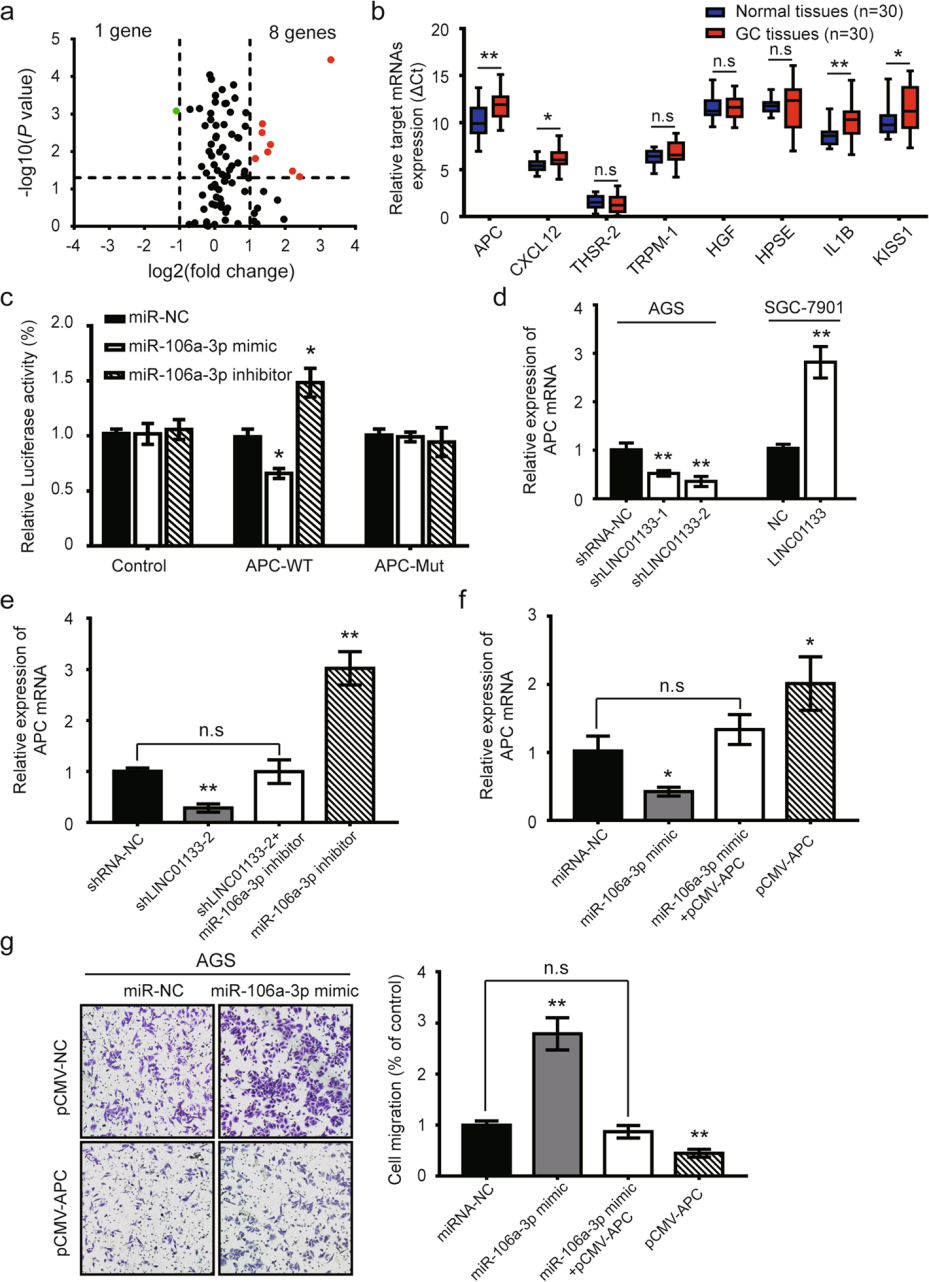
APC is a direct target of miR-106a-3p in GC metastasis. **a** Different expression of metastasis-related genes were examined in AGS cells after miR-106a-3p inhibitor or negative control transfection using a human tumor metastasis PCR array. Red marks, > 2-fold change (log2) and *P* <  0.05 (−log10); Green mark, < − 2-fold change (log2) and *P* <  0.05 (−log10). **b** The expression levels of eight upregulated candidate mRNAs confirmed by qRT-PCR in 30 paired GC cancer tissues and matched normal tissues. **c** Luciferase reporter assay in HEK-293FT cells co-transfected with wide type (WT) or mutated (Mut) APC 3’-UTR reporter vector and miR-106a-3p mimic or inhibitor. **d** Relative expression of APC mRNA in AGS cells with LINC01133 knockdown or SGC-7901 cells with LINC01133 overexpression. **e** qRT-PCR was conducted to evaluate the mRNA expression of APC gene in AGS cells following reduced expression of LINC01133 and/or inhibition of miR-106a-3p. **f** qRT-PCR was performed to access the mRNA expression of APC gene in AGS cells following ectopic expression of miR-106a-3p and/or pCMV-APC expression vector lacking the 3’-UTR. **g** Representative images and quantification of cell migration in AGS cells after transfection miR-106a-3p mimic and/or pCMV-APC lacking the 3’-UTR. Error bars: mean ± SD, *n* = 3. ^*^P <  0.05 and ^**^P < 0.01

miRNAs exert their multiple biological functions mainly by suppressing mRNA translation or degrading mRNA [7]. We further examined the differential expression of metastasis-related genes using a human tumor metastasis PCR array. Eight upregulated mRNAs and only one downregulated mRNA were found in AGS-miR-106a-3p inhibitor cells (Fig. 5a). In the eight upregulated mRNAs, we focused on the APC gene, the significantly downregulated mRNA, as the primary candidate (Fig. 5b). The alignment of complementary binding of miR-106a-3p and 3’-UTR of APC was confirmed by the luciferase assay (Fig. 5c and Additional file 5: Figure S3e). Then we clarified whether the APC gene was also modulated by the aberrant expression of LINC01133. Knockdown or overexpression of LINC01133 decreased or increased the expression of APC mRNA, respectively (Fig. 5d). Consistently, miR-106a-3p inhibitor could partially rescue the inhibitory effect of LINC01133 on APC expression (Fig. 5e). Similarly, AGS cells co-transfected with miR-106a-3p mimic and pCMV-APC also partially restored the loss of APC gene in cells transfected with miR-106a-3p mimic only (Fig. 5f). A similar phenomenon was observed in transwell assay experiments (Fig. 5g). These data provide evidence that the role of LINC01133 in suppressing GC metastasis is primarily dependent on the miR-106a-3p/APC axis.

### LINC01133/miR-106a-3p/APC axis negatively regulates metastasis and EMT via Wnt/β-cantenin signaling

**Fig. 6:**
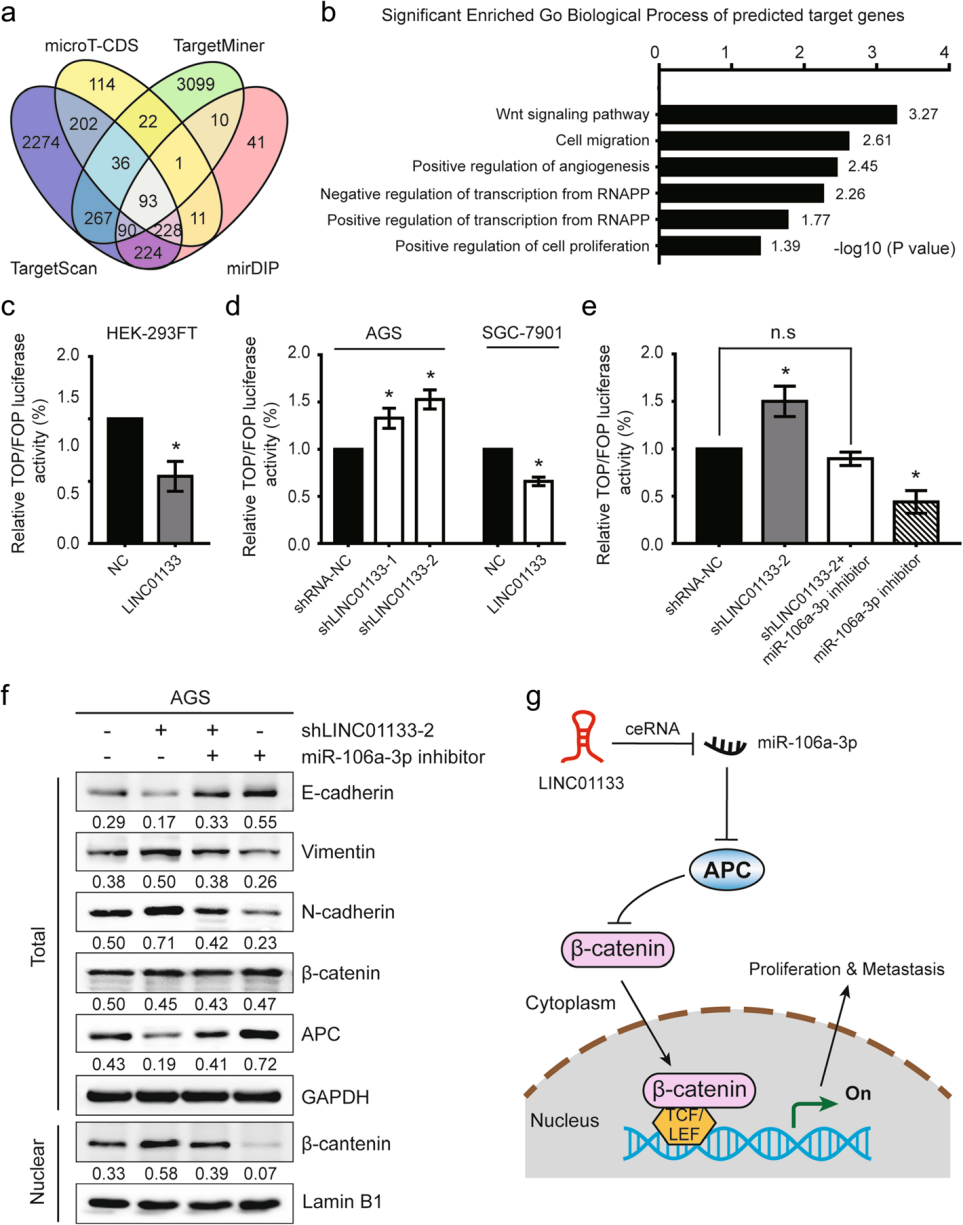
LINC01133 attenuates the EMT and metastatic abilities of GC cells through APC/Wnt signaling pathway. **a** Identification of 93 commonly changed targeted mRNAs of miR-106a-3p from the four publicly profile datasets (TargetScan, microT-CDS, TargetMiner, and mirDIP). Different color areas represented different datasets. The cross areas meant the commonly changed mRNAs of miR-106a-3p. **b** GO analysis and significant enriched GO terms of 93 commonly changed targeted mRNAs in gastric cancer on their biological process (BP). The statistically significant results were defined with -log10 (*P* value) > 1.30 as the cut-off criterion. **c**, **d** Dual luciferase assay demonstrating the effect on TOP/FOP reporter activity in HEK-293FT cells, AGS cells transfected with shLINC01133 vector or SGC-7901 cells with LINC01133 overexpression. Results were normalized to a Renilla transfection control. **e** Dual luciferase assay showing the effect on TOP/FOP reporter activity in AGS cells following reduced expression of LINC01133 and/or inhibition of miR-106a-3p. **f** Immunoblot assay of E-cadherin, vimentin, N-cadherin, APC, and total and nuclear β-catenin proteins in AGS cells transfected with shLINC01133–2 and/or miR-106a-3p inhibitor. Numbers showed quantification of relative protein amount. GAPDH was used as an internal control. Lamin B1 was used as an endogenous control for the cell nuclear fraction. **g** Schematic diagram of the regulatory mechanism of LINC01133/miR-106a-3p/APC axis in the inhibition of GC proliferation and metastasis. Error bars: mean ± SD, *n* = 3. ^*^P < 0.05 versus corresponding control

To determine whether the signaling pathways are involved in LINC01133/miR-106a-3p regulation of GC metastasis, we performed integrated bioinformatical analysis and found that 93 mRNAs were the potential targets of miR-106a-3p (Fig. 6a), which also contained APC gene. Subsequently, we predicted the biological processes of 93 potential target mRNAs using the online database DAVID 6.7, with *P* <  0.05 as the cut-off criterion. Gene Ontology (GO) biological process enrichment analysis demonstrated that these candidate genes were mainly enriched in Wnt signaling pathway and cell migration (Fig. 6b), which was consistent with the in vitro results. Thus, TOPFlash and FOPFlash reporters, containing wild-type and mutated TCF-4 consensus binding sites respectively, were constructed to verify whether LINC01133/miR-106a-3p modulates the canonical Wnt pathway by regulating APC. As expected, stably expressed LINC01133 in HEK-293FT and SGC-7901 cells remarkably decreased TOP/FOP transcriptional activity (Fig. 6c, d), whereas TOP/FOP activity was increased when LINC01133 was downregulated in AGS cells (Fig. 6d). Further analysis showed an obvious increase in luciferase activity in the absence of LINC01133 was partially reversed by inhibiting miR-106a-3p (Fig. 6e). The abovementioned data indicate that LINC01133/miR-106a-3p inactivates the Wnt signaling pathway in GC cells.

It has been well established that miRNAs play key roles in regulating the EMT process in GC through Wnt signaling [26, 27]. Therefore, we observed that abnormal expression of EMT-related proteins induced by LINC01133 knockdown was reversed after introduction of a miR-106a-3p inhibitor (Fig. 6f). Likewise, the inhibitory role of LINC01133 on APC expression was restored by co-transfection with miR-106a-3p inhibitor, whereas this co-transfection did not influence the total expression of β-catenin (Fig. 6f). However, when LINC01133 was knocked down, the nuclear expression of β-catenin was dramatically increased (Fig. 6f). The ratio of nuclear/cytoplasmic expression of β-catenin protein in AGS cells with LINC01133 knockdown was nearly 10 times higher than in cells with miR-106a-3p inhibition (1.3 vs. 0.14, data not shown), but it was contracted after co-transfected with miR-106a-3p inhibitor (1.3 vs. 0.92, data not shown) (Fig. 6f). These data implied that LINC01133 inhibited the nuclear accumulation of β-catenin protein via suppressing miR-106a-3p and promoting APC expression. Overall, our data presented suggest that LINC01133/miR-106a-3p can block the nuclear accumulation of β-catenin protein, and the LINC01133/miR-106a-3p/APC axis regulates GC cell metastasis in a Wnt-dependent manner.

## Discussion

Recently, several lncRNAs have been found to be dysregulated in gastrointestinal carcinoma including GC [28, 29]. The proverbial lncRNA HOTAIR can accelerate the EMT process and GC migration by activating the C-Met/Snail pathway via repressing PRC2/miR34a [30]. HOTAIR also enhanced the chemoresistant ability of GC cells by regulating PI3K/Akt and Wnt/β-catenin signaling pathways [31]. In this study, we demonstrated that LINC01133 was significantly decreased in GC tissues and cell lines, and LINC01133 inhibited tumor growth, the EMT process, and metastasis both in vitro and in vivo, suggesting that LINC01133 can act as a tumor suppressor in GC. Consequently, LINC01133 may serve a potential diagnostic and prognostic biomarker for improving GC patients’ outcome.

An increasing number of studies have highlighted that different biological functions of lncRNAs largely depend on their distinct subcellular localization [32]. Cytoplasmic lncRNAs can regulate mRNA stability or translation and influence signaling pathways by acting as decoys for miRNAs [25]. Using bioinformatic analyses and luciferase reporter assays, we demonstrated that LINC01133 directly binds and inhibits miRNA-106a-3p expression. Although miR-106a and miR-106a-5p are key regulators of human cancer development and progression [33–36], no research studies on the function and mechanism of miR-106a-3p have been conducted to date. Our study provides the first evidence that miRNA-106a-3p expression is significantly upregulated and inversely correlated with LINC01133 expression in GC. Overexpression of miRNA-106a-3p prevented the anti-metastatic ability of LINC01133. Zeng et al. [13] reported that LINC01133 acts as a specifically molecular sponge for miR-422a in human osteosarcoma. A similar ceRNA mechanism in another cancer type confirmed our findings in GC.

Recent discoveries have revealed that miRNAs act as critical signal transduction mediators in cancer signaling pathways by degrading or inhibiting mRNAs, thereby influencing cell fate and function [7]. The EMT and its associated Wnt/β-catenin pathway have recently been clarified as crucial drivers of embryonic development and carcinoma metastasis [37, 38]. Growing evidence has pointed out that miRNAs modulated the EMT by interacting with certain target mRNAs of the Wnt/β-catenin pathway in GC [39]. Zhang et al. [23] demonstrated that miR-27 promoted the EMT and GC metastasis by directly targeting APC to activate Wnt pathway. The miRNA-200 family hindered the EMT through upregulating ZEB1/2 and impacting E-cadherin/β-catenin expression in GC [26, 40]. In the current study, we also found that the APC gene is a direct target of miR-106a-3p. Further analysis showed that inhibition of miR-106a-3p significantly decreased TOP/FOP activity, indicating that the Wnt/β-catenin pathway was inhibited. However, TOP/FOP activity was increased in cells co-transfected with miR-106a-3p inhibitor and shLINC01133. Except for the APC gene, three mRNAs (CXCL12, IL1B and KISS1) were also significantly down-regulated in GC samples. However, bioinformatics analysis indicated that none of them had a potential binding site for miR-106a-3p (data not shown), suggesting the specificity of miR-106a-3p binding to APC gene in GC. Therefore, our results suggest that the LINC01133/miR-106a-3p axis can activate the canonical Wnt/β-catenin signaling pathway by specifically suppressing APC expression.

Interestingly, a recent study demonstrated that the C/EBPβ transcription factor could bind the promoter of LINC01133 and upregulated the expression of LINC01133 in pancreatic ductal adenocarcinoma [41]. However, the mechanism underlying the downregulation of LINC0133 in GC is still largely unknown, and it remains unclear whether certain transcription factors also regulate LINC01133 downregulation in GC. Besides, considering previous studies in CRC, lung cancer and pancreatic cancer [9, 14, 41], whether LINC01133 might bind certain proteins or regulate other pathways to exert its inhibitory effect on GC progression remains mysterious. We plan to further investigate these issues in our future studies.

## Conclusions

In summary, our data indicated that decreased LINC01133 expression is a common event and an independent prognostic biomarker in GC. LINC01133 functioned as an anti-proliferative and anti-metastatic lncRNA in GC both in vitro and in vivo. Our study defines a mechanism for the suppressor function of LINC01133 that directly targets miR-106a-3p, in turn, inhibits GC metastasis through inactivating the APC/Wnt/β-catenin pathway (Fig. 6g). Therefore, LINC01133 may serve as a novel prognostic biomarker and a potential anti-metastatic therapeutic target for GC treatment.

## Additional files

Additional file 1: **Table S1.** Primers and oligonucleotides sequences used in this study. (DOCX 15 kb)

Additional file 2: **Figure S1.** The expression of LINC01133 in GC patents with different clinical subgroups. a-d Relative expression of LINC01133 in GC with different tumor sizes, with/without infiltration of peritumoral tissues (IPT), with/without distant metastasis, and with/without peritoneum dissemination (PD). Results were presented as Δ cycle threshold (ΔCt) in tumor tissues relative to normal tissues. e qRT-PCR was performed to examine LINC01133 expression in 200 GC cancer tissues. Relative expression of LINC01133 was presented as log2 (fold change of ΔCt value) in tumor tissue to that of matched normal tissues. GC patients were divided into high (*n* = 100) and low (*n* = 100) groups according to the median value (0.50). (TIF 819 kb)

Additional file 3: **Table S2.** Correlation between LINC01133 expressions and clinicopathological parameters in gastric cancer (DOCX 18 kb)

Additional file 4: **Figure S2.** LINC01133 knockdown enhances GC cells motility and LINC01133 overexpression inhibits colony conformation in vitro and Ki-67 and MMP-9 expression in vivo. a Wound healing assay were performed to determine cell motility of shLINC01133-transfected AGS and BGC-823 cells. Quantifications were shown in right histogram. b Colony formation assay of SGC-7901 and HGC-27 cells with stable expression of LINC01133. Representative results and quantifications were shown. Data (mean ± SD, *n* = 3) were analyzed by Student *t* test; ^*^P <  0.05. c H&E and IHC staining of Ki-67 and MMP-9 proteins in xenograft tumors. Scale bars: 50 μm. d qRT-PCR was used to detect the relative expressions of Ki-67 and MMP-9 genes in lung metastases originated from mice in LINC01133 overexpression groups and control group. The results are shown as the mean ± SD, *n* = 3. ^*^P <  0.05 and ^**^P <  0.01. (TIF 8392 kb)

Additional file 5: **Figure S3.** Predicted target miRNAs of LINC01133 and predicted binding sites for miR-106a-3p in LINC01133 or APC gene. a FISH detection for LINC01133 (red) was performed in AGS cells. The nucleus was counterstained with DAPI (blue). Scale bar = 10 μm. (b) Identification of 162 predicted target miRNAs of LINC01133 from five publicly bioinformatic databases (lncRNAMap, LNCipedia, miRcode, LncBase Predicted, and LncBase Experimental). Different color areas represented different datasets. c Relative expressions of miR-106a-3p examined by qRT-PCR in 200 paired GC cancer tissues and matched normal tissues. Results were presented as Δ cycle threshold (ΔCt) in tumor tissues relative to normal tissues. d Schematic representation of two predicted binding sites for miR-106a-3p in LINC01133 by online database LncBase Predicted algorithm. The numbers indicate the positions of the nucleotides in the reference wild-type sequence of LINC01133 (Ensembl version: ENSG00000224259). e Schematic representation of the predicted miR-106a-3p target site within the 3′-UTR of APC. The predicted target site for miR-106a-3p is located at the proximal portion of the APC 3′-UTR. Two nucleotides complementary to the seed sequence (the nucleotides 2–7 of miRNA) of miR-106a-3p were mutated in the APC mutant plasmid. The number indicates the position of the nucleotides in the reference wild-type sequence of APC (NM_000038.5). (TIF 1168 kb)
