# Supplementary material for: LINC01133 as ceRNA inhibits gastric cancer progression by sponging miR-106a-3p to regulate APC expression and the Wnt/β-catenin pathway
Source: Mol Cancer. 2018 Aug 22;17:126. doi: 10.1186/s12943-018-0874-1 (PMC6106894; doi:10.1186/s12943-018-0874-1)
Supplement: Supplementary file 1 — Table S1. Primers and oligonucleotides sequences used in this study. (DOCX 15 kb) [file 12943_2018_874_MOESM1_ESM.docx]

**Table S1.** Primers and oligonucleotides sequences used in this study

| Name | Sequence 5'-3' |
| --- | --- |
| LINC01133-forward | TGGTGGAGAGAATGGAGG |
| LINC01133-reverse | AACCCAGTTCCTTAGAATCTTC |
| LINC01133-shRNA-1-forward | CCGGAACCAGAAAUACUUAAUUCAACTCGAGTTGTTGTTTTCTGGTTTTTTTG |
| LINC01133-shRNA-1-reverse | AATTCAAAAAAACCAGAAAUACUUAAUUCAACTCGAGTTGTTGTTTTCTGGTT |
| LINC01133-shRNA-2-forward | CACCAATGGATCCATTCCCTGCAACTGAACGAATTCAGTTGCAGGGAATGGATCCA |
| LINC01133-shRNA-2-reverse | CACCGAAGTGGAAGCAAAGTTCTCCAAAGCGAACTTTGGAGAACTTTGCTTCCACTTC |
| LINC01133-FISH probe | CAAGAGGAGAAAGCCAGGGACT |
| Control-FISH probe | GTTCTCCTCTTTCGGTCCCTGA |
| APC-forward | AAAATGTCCCTCCGTTCTTATGG |
| APC-reverse | CTGAAGTTGAGCGTAATACCAGT |
| CXCL12-forward | ATTCTCAACACTCCAAACTGTGC |
| CXCL12-reverse | ACTTTAGCTTCGGGTCAATGC |
| TSHR-forward | TTCCCTGACCTGACCAAAGTT |
| TSHR-reverse | ACGTCATGTAAGGGTTGTCTGT |
| TRPM1-forward | GTTCACCAACCAGCATATCCC |
| TRPM1-reverse | GCTTTATTGGAATATCCGCCACC |
| HGF-forward | GCTATCGGGGTAAAGACCTACA |
| HGF-reverse | CGTAGCGTACCTCTGGATTGC |
| HPSE-forward | TCATCAATGGGTCGCAGTTAGG |
| HPSE-reverse | TTAGCCGTCTTTCTTCGAGGC |
| IL1B-forward | ATGATGGCTTATTACAGTGGCAA |
| IL1B-reverse | GTCGGAGATTCGTAGCTGGA |
| KISS1-forward | AGCAGCTAGAATCCCTGGG |
| KISS1-reverse | AGGCCGAAGGAGTTCCAGT |
| Ki-67-forward | AGAAGAAGTGGTGCTTCGGAA |
| Ki-67-reverse | AGTTTGCGTGGCCTGTACTAA |
| MMP9-forward | GGGACGCAGACATCGTCATC |
| MMP9-reverse | TCGTCATCGTCGAAATGGGC |
| GAPDH-forward | CTCCTCCTGTTCGACAGTCAGC |
| GAPDH-reverse | CCCAATACGACCAAATCCGTT |
