# Supplementary figures and images for: LINC01133 as ceRNA inhibits gastric cancer progression by sponging miR-106a-3p to regulate APC expression and the Wnt/β-catenin pathway
Source: Mol Cancer. 2018 Aug 22;17:126. doi: 10.1186/s12943-018-0874-1 (PMC6106894; doi:10.1186/s12943-018-0874-1)

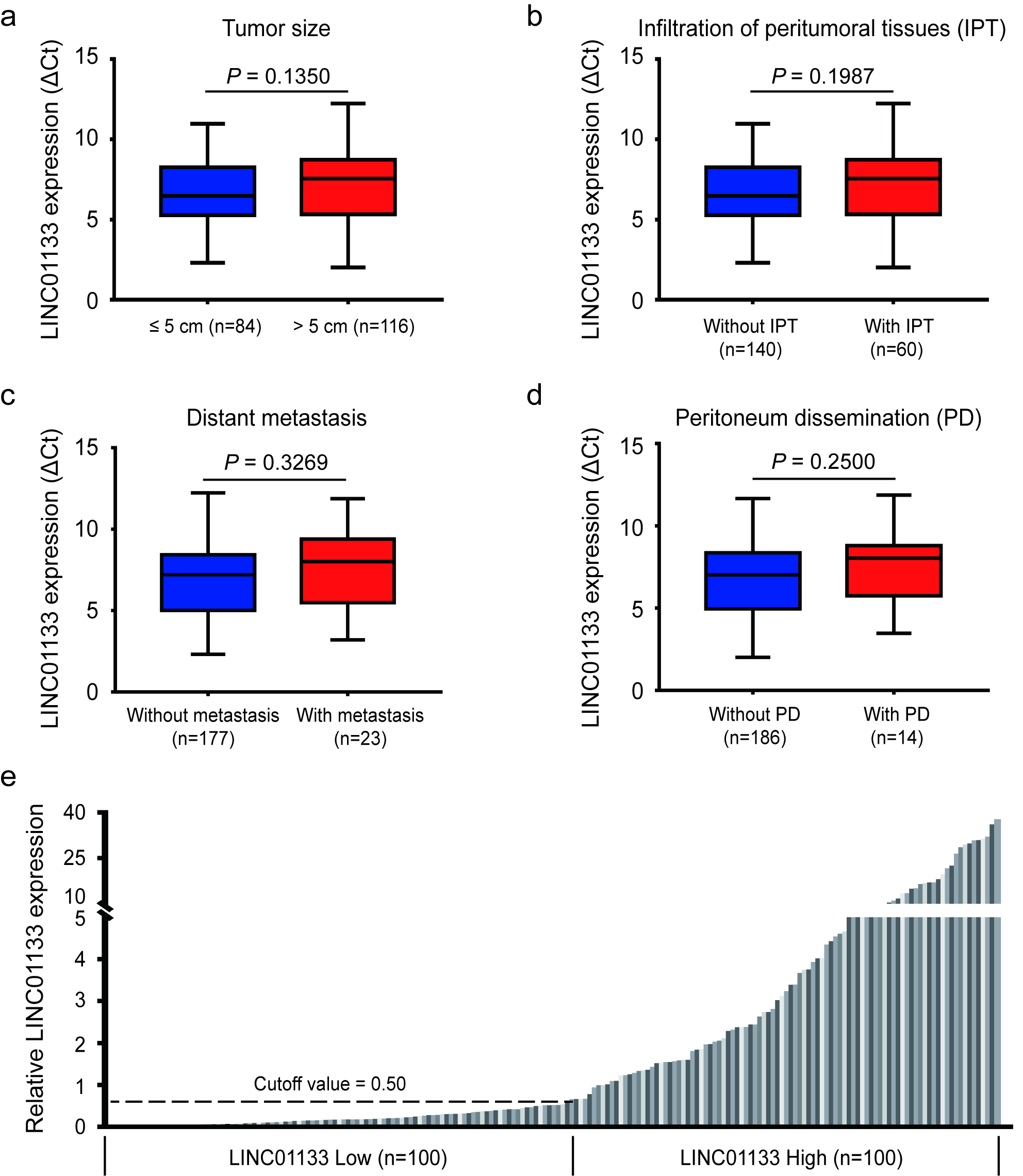

Supplement: Supplementary file 2 — Figure S1. The expression of LINC01133 in GC patents with different clinical subgroups. a-d Relative expression of LINC01133 in GC with different tumor sizes, with/without infiltration of peritumoral tissues (IPT), with/without distant metastasis, and with/without peritoneum dissemination (PD). Results were presented as Δ cycle threshold (ΔCt) in tumor tissues relative to normal tissues. e qRT-PCR was performed to examine LINC01133 expression in 200 GC cancer tissues. Relative expression of LINC01133 was presented as log2 (fold change of ΔCt value) in tumor tissue to that of matched normal tissues. GC patients were divided into high (n = 100) and low (n = 100) groups according to the median value (0.50). (TIF 819 kb) [file 12943_2018_874_MOESM2_ESM.tif]

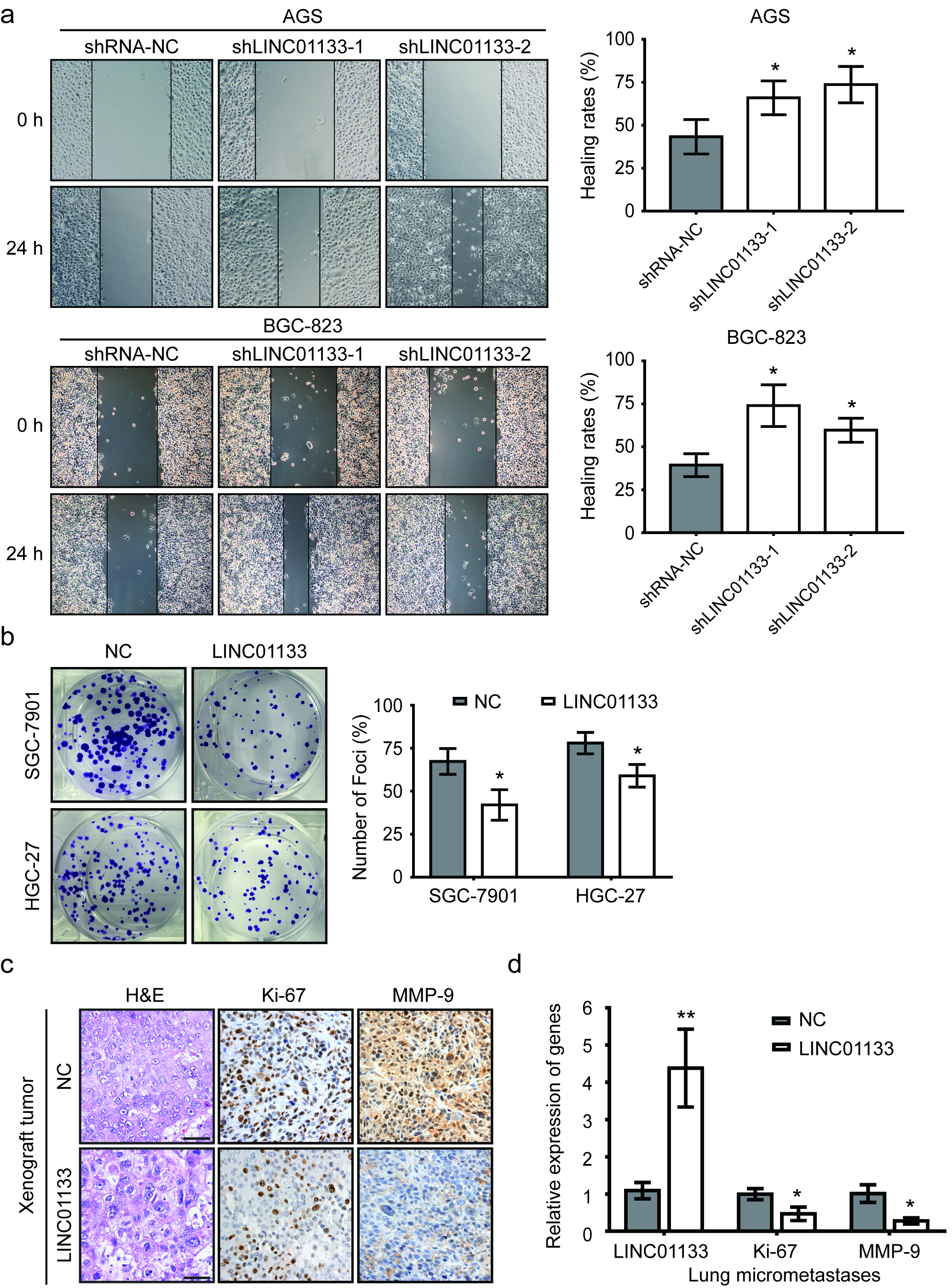

Supplement: Supplementary file 4 — Figure S2. LINC01133 knockdown enhances GC cells motility and LINC01133 overexpression inhibits colony conformation in vitro and Ki-67 and MMP-9 expression in vivo. a Wound healing assay were performed to determine cell motility of shLINC01133-transfected AGS and BGC-823 cells. Quantifications were shown in right histogram. b Colony formation assay of SGC-7901 and HGC-27 cells with stable expression of LINC01133. Representative results and quantifications were shown. Data (mean ± SD, n = 3) were analyzed by Student t test; *P < 0.05. c H&E and IHC staining of Ki-67 and MMP-9 proteins in xenograft tumors. Scale bars: 50 μm. d qRT-PCR was used to detect the relative expressions of Ki-67 and MMP-9 genes in lung metastases originated from mice in LINC01133 overexpression groups and control group. The results are shown as the mean ± SD, n = 3. *P < 0.05 and **P < 0.01. (TIF 8392 kb) [file 12943_2018_874_MOESM4_ESM.tif]

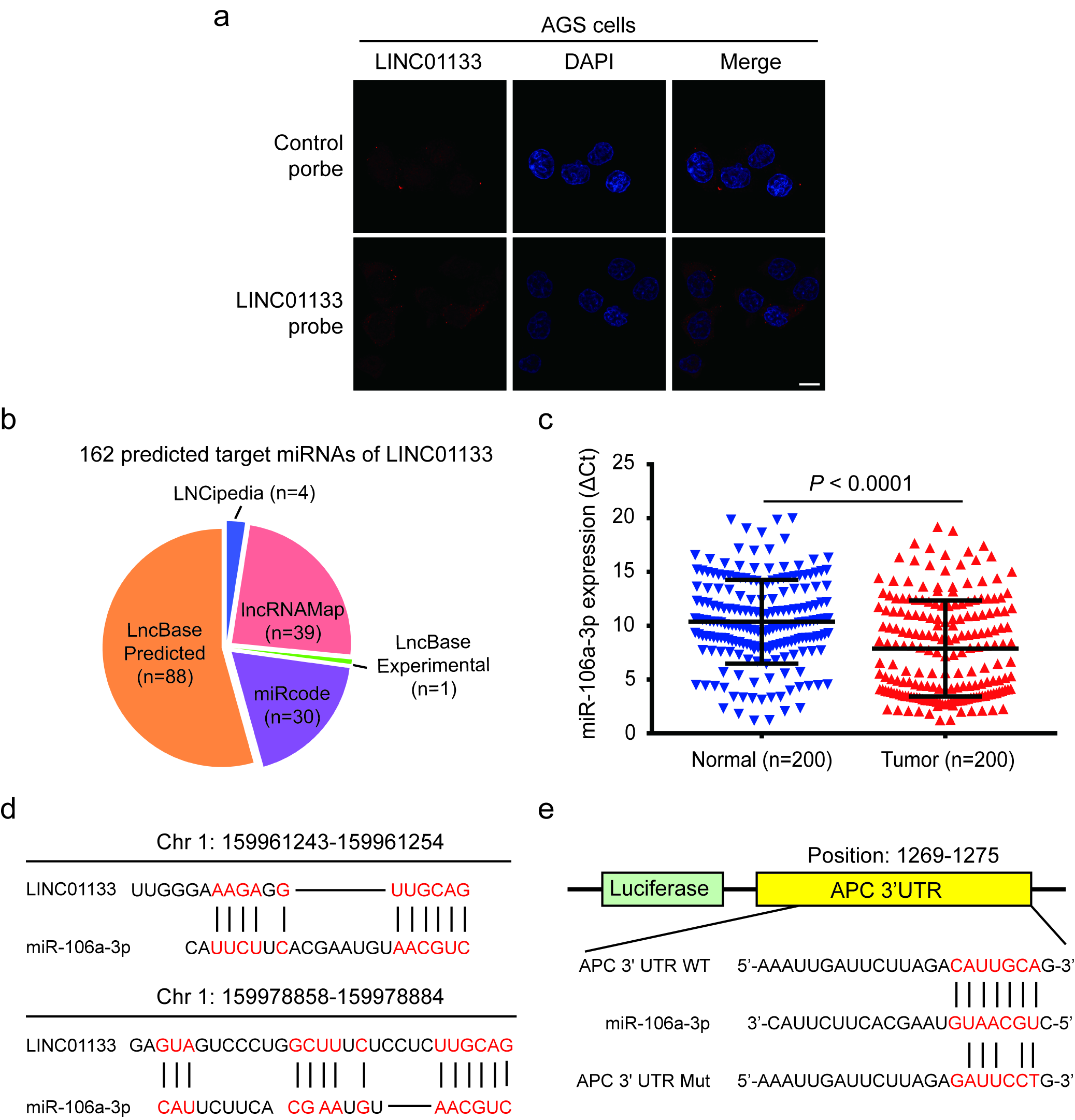

Supplement: Supplementary file 5 — Figure S3. Predicted target miRNAs of LINC01133 and predicted binding sites for miR-106a-3p in LINC01133 or APC gene. a FISH detection for LINC01133 (red) was performed in AGS cells. The nucleus was counterstained with DAPI (blue). Scale bar = 10 μm. (b) Identification of 162 predicted target miRNAs of LINC01133 from five publicly bioinformatic databases (lncRNAMap, LNCipedia, miRcode, LncBase Predicted, and LncBase Experimental). Different color areas represented different datasets. c Relative expressions of miR-106a-3p examined by qRT-PCR in 200 paired GC cancer tissues and matched normal tissues. Results were presented as Δ cycle threshold (ΔCt) in tumor tissues relative to normal tissues. d Schematic representation of two predicted binding sites for miR-106a-3p in LINC01133 by online database LncBase Predicted algorithm. The numbers indicate the positions of the nucleotides in the reference wild-type sequence of LINC01133 (Ensembl version: ENSG00000224259). e Schematic representation of the predicted miR-106a-3p target site within the 3′-UTR of APC. The predicted target site for miR-106a-3p is located at the proximal portion of the APC 3′-UTR. Two nucleotides complementary to the seed sequence (the nucleotides 2–7 of miRNA) of miR-106a-3p were mutated in the APC mutant plasmid. The number indicates the position of the nucleotides in the reference wild-type sequence of APC (NM_000038.5). (TIF 1168 kb) [file 12943_2018_874_MOESM5_ESM.tif]
