# Supplementary material for: LINC01133 as ceRNA inhibits gastric cancer progression by sponging miR-106a-3p to regulate APC expression and the Wnt/β-catenin pathway
Source: Mol Cancer. 2018 Aug 22;17:126. doi: 10.1186/s12943-018-0874-1 (PMC6106894; doi:10.1186/s12943-018-0874-1)
Supplement: Supplementary file 3 — Table S2. Correlation between LINC01133 expressions and clinicopathological parameters in gastric cancer (DOCX 18 kb) [file 12943_2018_874_MOESM3_ESM.docx]

| **Table S2**. Correlation between LINC01133 expressions and clinicopathological parameters in gastric cancer | | | | |
| --- | --- | --- | --- | --- |
| Clinical variables | N | LINC01133 Expression | | *P* value |
|  |  | Low (N, %) | High (N, %) |  |
| Gender |  |  |  |  |
| Female | 63 | 37 (58.7%) | 26 (41.3%) | 0.138 |
| Male | 137 | 65 (47.4%) | 72 (52.6%) |  |
| Age (years) |  |  |  |  |
| < 60 | 113 | 57 (50.4%) | 56 (49.6%) | 0.857 |
| ≥ 60 | 87 | 45 (51.7%) | 42 (48.3%) |  |
| Tumor location |  |  |  |  |
| Upper | 54 | 32 (59.3%) | 22 (40.7%) | 0.135 |
| Middle | 52 | 29 (55.8%) | 23 (44.2%) |  |
| Down | 94 | 41 (43.6%) | 53 (56.4%) |  |
| Tumor size |  |  |  |  |
| < 5 cm | 84 | 35 (41.7%) | 49 (58.3%) | **0.025** |
| ≥ 5 cm | 116 | 67 (57.8%) | 49 (42.2%) |  |
| Differentiation |  |  |  |  |
| Well | 1 | 1 (100.0%) | 0 (0.0%) | 0.592 |
| Moderate | 33 | 16 (48.5%) | 17 (51.5%) |  |
| Poor | 166 | 85 (51.2%) | 81 (48.8%) |  |
| T stage |  |  |  |  |
| T1 | 10 | 2 (20.0%) | 8 (80.0%) | **0.011** |
| T2 | 17 | 4 (23.5%) | 13 (76.5%) |  |
| T3 | 44 | 27 (61.4%) | 17 (38.6%) |  |
| T4 | 129 | 69 (53.5%) | 60 (46.5%) |  |
| N stage |  |  |  |  |
| N0 | 42 | 14 (33.3%) | 28 (66.7%) | **0.015** |
| N1 | 32 | 13 (40.6%) | 19 (59.4%) |  |
| N2 | 47 | 27 (57.4%) | 20 (42.6%) |  |
| N3 | 79 | 48 (60.8%) | 31 (39.2%) |  |
| TNM stage |  |  |  |  |
| I | 13 | 4 (30.8%) | 9 (69.2%) | **0.005** |
| II | 48 | 16 (33.3%) | 32 (66.7%) |  |
| III | 116 | 66 (56.9%) | 50 (43.1%) |  |
| IV | 23 | 16 (69.6%) | 7 (30.4%) |  |
| Distant metastasis | | | | |
| No | 177 | 86 (48.6%) | 91 (51.4%) | 0.058 |
| Yes | 23 | 16 (69.6%) | 7 (30.4%) |  |
| Infiltration of peritumoral tissues | | | | |
| No | 140 | 63 (45.0%) | 77 (55.0%) | **0.010** |
| Yes | 60 | 39 (65.0%) | 21 (35.0%) |  |
| Peritoneum dissemination | | | | |
| No | 186 | 92 (49.5%) | 94 (50.5%) | 0.113 |
| Yes | 14 | 10 (71.4%) | 4 (28.6%) |  |
| NOTE: TNM, tumour node metastasis. The bold type represents *P* values smaller than 0.05. | | | | |
